# Comparative anatomy of dissected optic lobes, optic ventricles, midbrain tectum, collicular ventricles, and aqueduct: evolutionary modifications as potential explanation for non-tumoral aqueductal anomalies in humans

**DOI:** 10.1007/s00381-021-05408-0

**Published:** 2021-11-23

**Authors:** E. Leon Kier, Vivek B. Kalra, Gerald J. Conlogue, Cristopher G. Filippi, Sanjay Saluja

**Affiliations:** 1 Yale University School of Medicine, PO Box 208042, New Haven, CT 06520-8042 USA; 2 Quinnipiac University, 275 Mount Carmel Ave, CT 06518 Hamden, USA

**Keywords:** Anatomy Comparative, Optic lobe, Nonmammalian, Mammals, Tectum Mesencephali, Cerebral aqueduct, Dissection

## Abstract

**Purpose:**

An extensive literature has postulated multiple etiologies for aqueductal stenosis. No publications were found, discussing that evolutionary modifications might explain aqueductal anomalies. This study’s objectives were to review the evolutionary modifications of vertebrates’ tectum structures that might explain human aqueduct anomalies. Undertaking vertebrate comparative study is currently not feasible in view of limitations in obtaining vertebrate material. Thus, vertebrate material collected, injected, dissected, and radiographed in the early 1970s was analyzed, focusing on the aqueduct and components of the midbrain tectum.

**Methods:**

Photographs of brain dissections and radiographs of the cerebral ventricles and arteries of adult shark, frog, iguana, rabbit, cat, dog, and primate specimens, containing a barium-gelatin radiopaque compound, were analyzed focusing on the aqueduct, the optic ventricles, the quadrigeminal plate, and collicular ventricles. The anatomic information provided by the dissections and radiographs is not reproducible by any other radiopaque contrast currently available.

**Results:**

Dissected and radiographed cerebral ventricular and arterial systems of the vertebrates demonstrated midbrain tectum changes, including relative size modifications of the mammalian components of the tectum, simultaneously with the enlargement of the occipital lobe. There is a transformation of pre-mammalian optic ventricles to what appear to be collicular ventricles in mammals, as the aqueduct and collicular ventricle form a continuous cavity.

**Conclusions:**

The mammalian tectum undergoes an evolutionary cephalization process consisting of relative size changes of the midbrain tectum structures. This is associated with enlargement of the occipital lobe, as part of overall neocortical expansion. Potentially, aqueductal anomalies could be explained by evolutionary modifications.

## Introduction

For over a century, an extensive literature has investigated the anatomy of the cerebral aqueduct, and explored the various potential etiologies of non-tumoral aqueductal stenosis [1, 24, 30]. Detailed MRI anatomy of the normal aqueduct has been described [20]. Advances in endoscopic exploration of the aqueduct, including photographs of its anomalies, and endoscopic treatment of aqueductal stenosis have been published [6, 7, 9, 18, 19, 26].

Evolutionary modifications of the midbrain tectum are not discussed in the extensive aqueductal stenosis literature. The objectives of this investigation were to review evolutionary material that contributed to the understanding of the evolutionary changes of the optic lobes and optic ventricles, the colliculi, the quadrigeminal plate of the midbrain tectum, and aqueduct. The comparative anatomic information may have the potential of pointing out that evolutionary modifications might explain the formation of forking, septa, and membranes resulting in aqueductal stenosis in humans.

## Material and methods

### Examination of the cerebral ventricular and arterial systems of various vertebrates

In the early 1970s, adult euthanized rabbit (*Lepus cuniculus*), cat (*Felis catus*), dog (*Canis familiaris*), and primate (*Macaca* sp) specimens were obtained from the ophthalmology, neurosurgery. cardiology, and gynecology research laboratories. The specimens of the dogfish shark (*Squalus acanthias*), American bullfrog (*Rana catesbeiana*), and green iguana (*Iguana iguana*) were obtained from biological material supply companies. These specimens were obtained as part of the material for studies of the evolutionary and embryologic changes of the brain, skull, spinal cord, and spine [14–17].

The cerebral ventricular system of several of these specimens was filled with a radiopaque barium-gelatin contrast agent, developed in the mid-1960s for use in specimen radiography [5]. It is based on a fine particle (0.1–0.4 μ) barium sulfate powder. In arterial injections, the small particulate size permits the barium to reach arterioles. For specimen radiography, in order to maintain the powder in suspension, mucilago acacia was added. To allow successful dissection, gelatin was added to the mixture. When protein, the basis of gelatin, is treated with a 10% buffered formalin, the protein remained solid, preventing the medium from returning to a liquid state. No other contrast media is available that is radiopaque, does not cause streaking artifacts on CT, micro-CT, and MRI imaging. In addition, it does not dissolve, shrink, and it remains within large or sub-millimeter structures, not only when dissected but also when subjected to sectioning.

The warm radiopaque barium-gelatin contrast agent was dripped into the unroofed fourth ventricle, followed by a downwards tilt of the front of the head in an effort to fill the entire cerebral ventricular system. Several of the specimens were also injected intra-arterially with the warm radiopaque barium-gelatin contrast agent.

Following the filling of the cerebral ventricular and arterial systems, fixation of the gelatin component of the contrast media was achieved by immersing the specimens in 10% buffered formalin for at least 10 days prior to dissection. Dissections were performed using a 3-diopter magnifying lamp and a dissecting microscope. Photography and plain radiography of the dissected specimens were performed.

### Fetal MRI study

Intermittently, during a number of years, co-authors VK, CF, and SS gathered midbrain tectum size data from human prenatal fetal MRI studies. Hand-drawn midbrain tectum and cerebellar vermis measurements of height and area were obtained from mid-sagittal T2-weighted images. The ratio of these measurements depicted progressive relative diminution of the tectal size in relation to the vermis.

However, limitation of the measurement techniques used, as well as the results of measurements of tectal height in photographs of midsagittal dissections of fetal specimens of increasing age, showed only a single mm difference in tectal height. The latter finding was not considered a significant change, in view of variation in estimated fetal age and specimen shrinkage in preservative solutions. As a result, the human fetal aspects were removed from the investigation to focus on the comparative anatomy of the midbrain tectum, as potentially, the evolutionary modifications may explain aqueductal anomalies such as forking, septa, and membranes in humans.

## Results

### Photographs of dissections and lateral radiographs demonstrate the contrast filled cerebral ventricular systems in various adult vertebrates

**Fig. 1:**
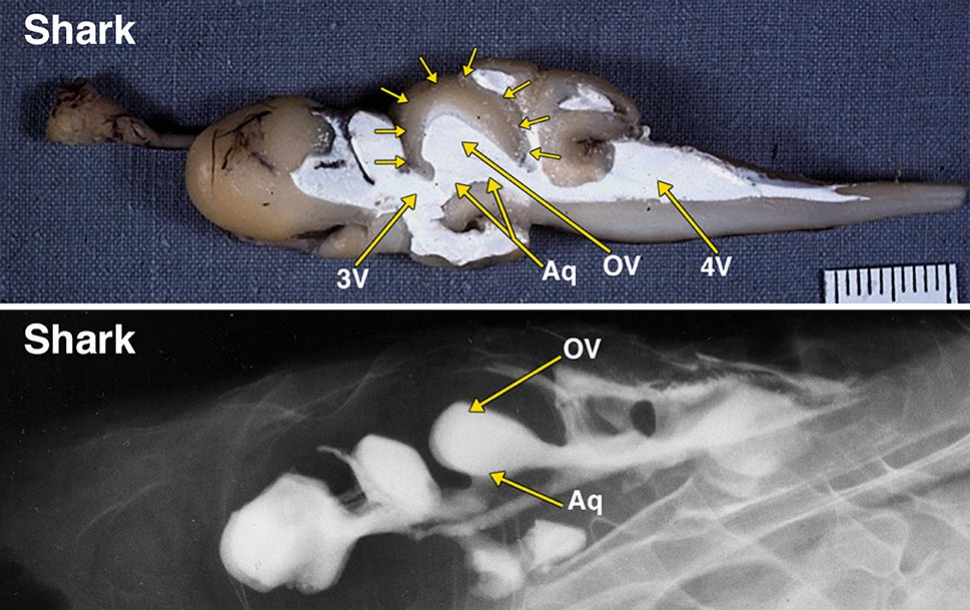
Dissected brain of a shark with radiopaque barium-gelatin-contrast filled cerebral ventricles. The midsagittal dissection (Fig. 1) demonstrates a relatively large, thin-walled optic lobe, outlined by multiple small arrows, that includes a large optic ventricle (OV). The midbrain ventricle (Aq) and optic ventricles form a large continuous cavity. The comparative anatomy literature uses the term aqueduct for the midbrain ventricle in all vertebrates with a cerebral ventricular system. The lateral radiograph of the entire dissected brain demonstrates both superimposed optic ventricles (OV) and the aqueduct (Aq). Additional abbreviations: 3 V, 3^d^ ventricle; 4 V, 4^th^ ventricle. The distance between 2 black lines on the ruler in the specimen photograph is 1 mm

**Fig. 2:**
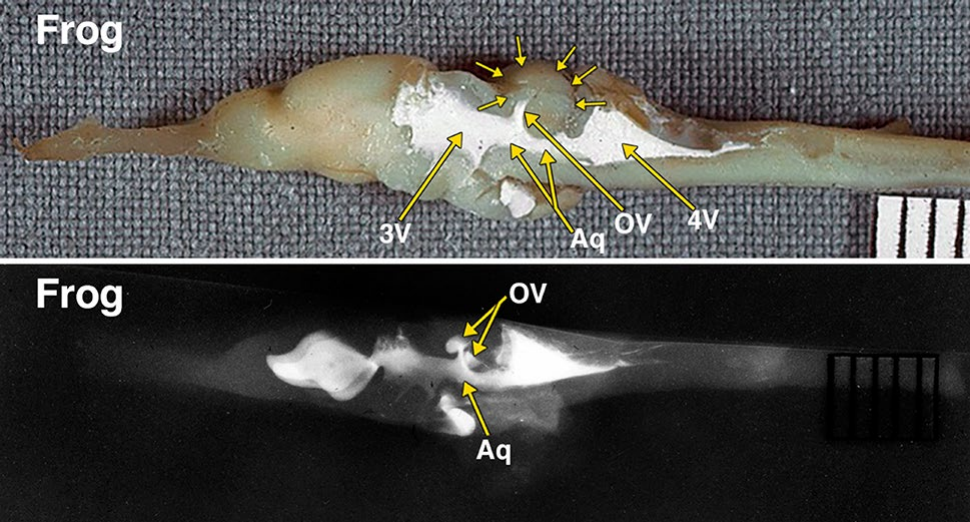
Dissected brain of a frog with radiopaque contrast filled cerebral ventricles. The midsagittal dissection (Fig. 2) demonstrates a prominent optic lobe, outlined by multiple small arrows, and includes only a small central segment of the optic ventricles (OV) extending superiorly from the aqueduct (Aq). The remainder of the more laterally positioned optic ventricles is visualized in the radiograph. The lateral radiograph of the entire dissected brain demonstrates both optic ventricles (OV) and the aqueduct (Aq). The constricted communication between the dorsolateral segment of the optic ventricles and medial segment of the opticventricle (aqueduct) is likely the result of compressed from below by the torus semicircularis [28]. Additional abbreviations: 3 V, 3^d^ ventricle; 4 V, 4^th^ ventricle. The distance between 2 black lines on the ruler in the specimen photograph is 1 mm

**Fig. 3:**
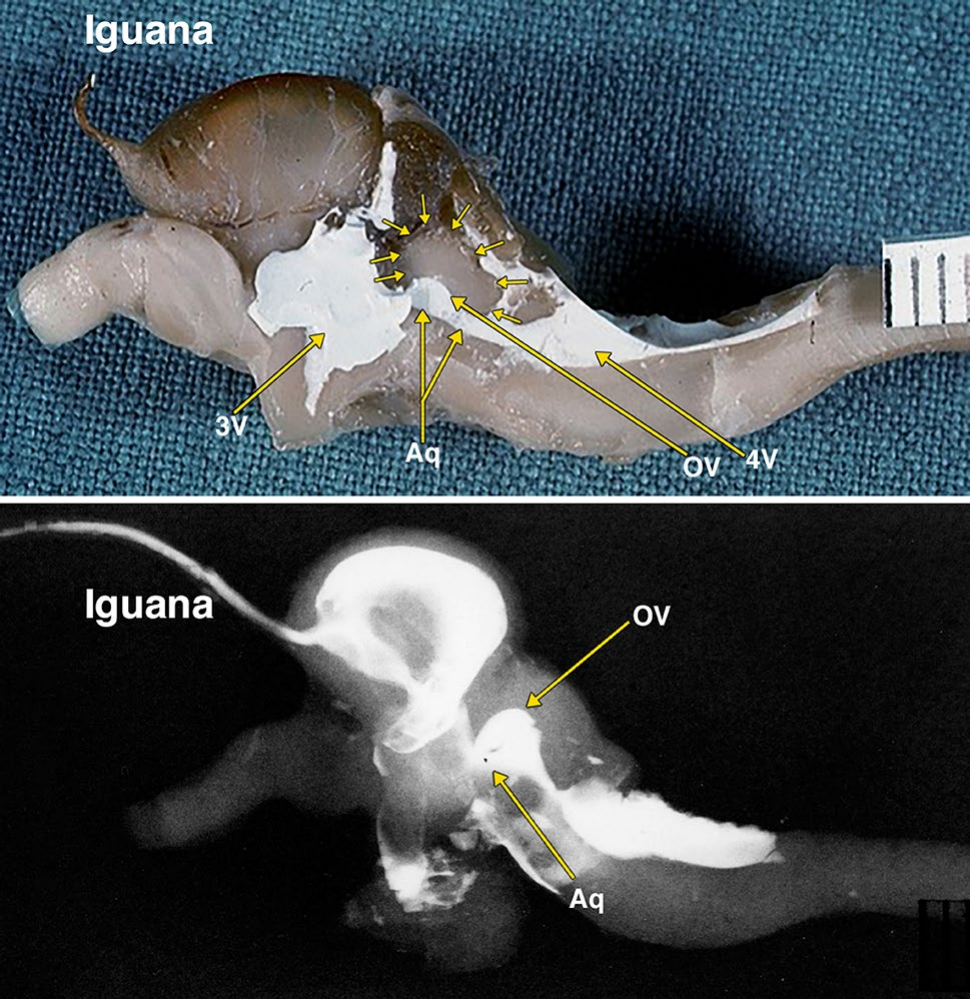
Dissected brain of an iguana with radiopaque contrast filled cerebral ventricles. The midsagittal dissection (Fig. 3) demonstrates a relatively large optic lobe, outlined by multiple small arrows, that includes an optic ventricle (OV). The midbrain ventricle (Aq) and optic ventricles form a large continuous cavity. The lateral radiograph (Fig. 3B) of the entire dissected brain demonstrates the mostly superimposed optic ventricles (OV) and the aqueduct (Aq). Additional abbreviations: 3 V, 3^d^ ventricle; 4 V, 4^th^ ventricle. The distance between 2 black lines on the ruler in the specimen photograph is 1 mm

**Fig. 4:**
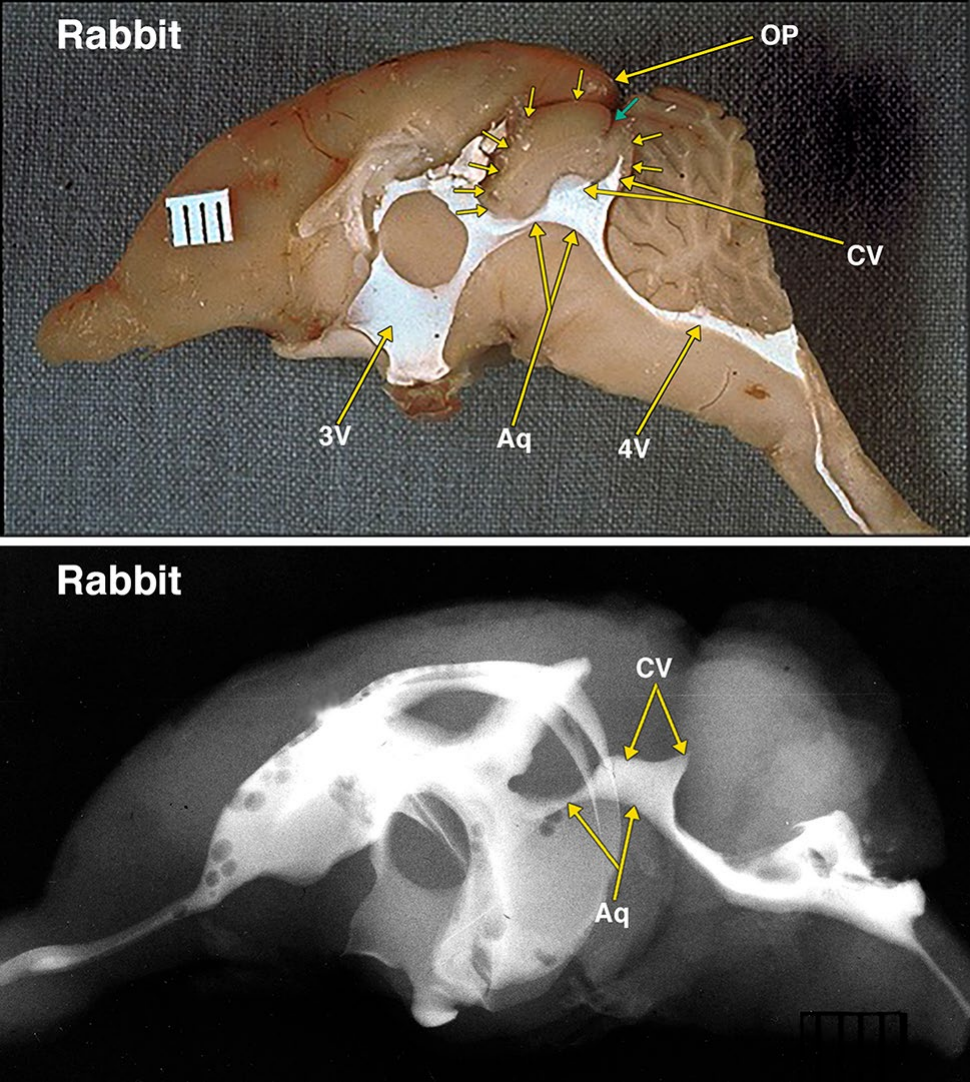
Dissected brain of a rabbit with radiopaque contrast filled cerebral ventricles. The midsagittal dissection (Fig. 4) demonstrates large midbrain colliculi outlined by multiple small arrows. A small cleft indicated by a small green arrow likely indicates the separate superior and inferior colliculi of the quadrigeminal plate. The aqueduct (Aq) and collicular ventricle (CV) form a large continuous cavity. Of note is the separate round extension of the collicular ventricle into what is likely the superior, and the pointed extension into the inferior colliculi. Also of note is the presence of a small neocortical occipital lobe and occipital pole (OP). The collicular ventricle features are also demonstrated in the lateral radiograph. Additional abbreviations: 3 V, 3^d^ ventricle; 4 V, 4^th^ ventricle. The distance between 2 black lines on the ruler in the specimen photograph is 1 mm

**Fig. 5:**
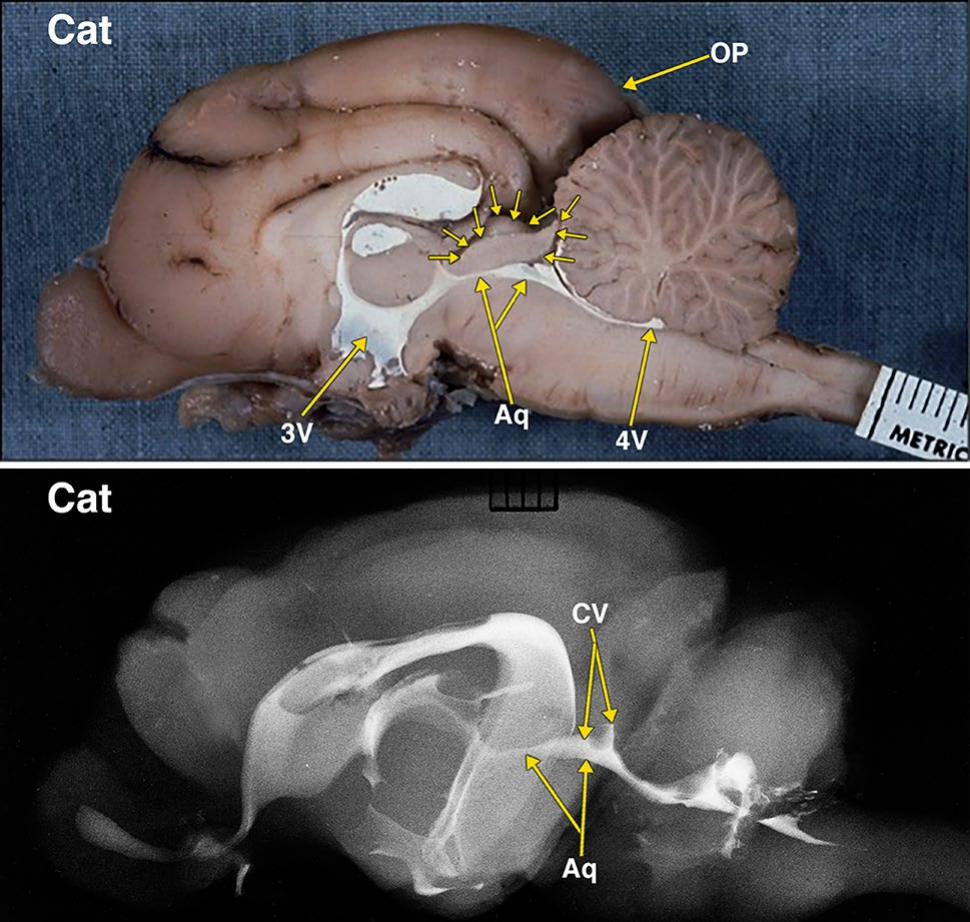
Dissected brain of a cat with radiopaque contrast filled cerebral ventricles. The midsagittal dissection (Fig. 5) demonstrates smaller midbrain colliculi, outlined by multiple small arrows, when compared with rabbit in Fig. 4. Of note is the presence of much larger neocortical occipital lobe and pole (OP) when compared to the rabbit in Fig. 4. The collicular ventricle (CV) superior extensions and its continuity with the midbrain aqueduct (Aq) features better demonstrated in the lateral radiograph. The collicular ventricle (CV) is more completely visualized on the radiograph as a result of the slight obliquity of the midsagittal plane of the anatomic dissection. Additional abbreviations: 3 V, 3^d^ ventricle; 4 V, 4^th^ ventricle. The distance between 2 black lines on the ruler in the specimen photograph is 1 mm

**Fig. 6:**
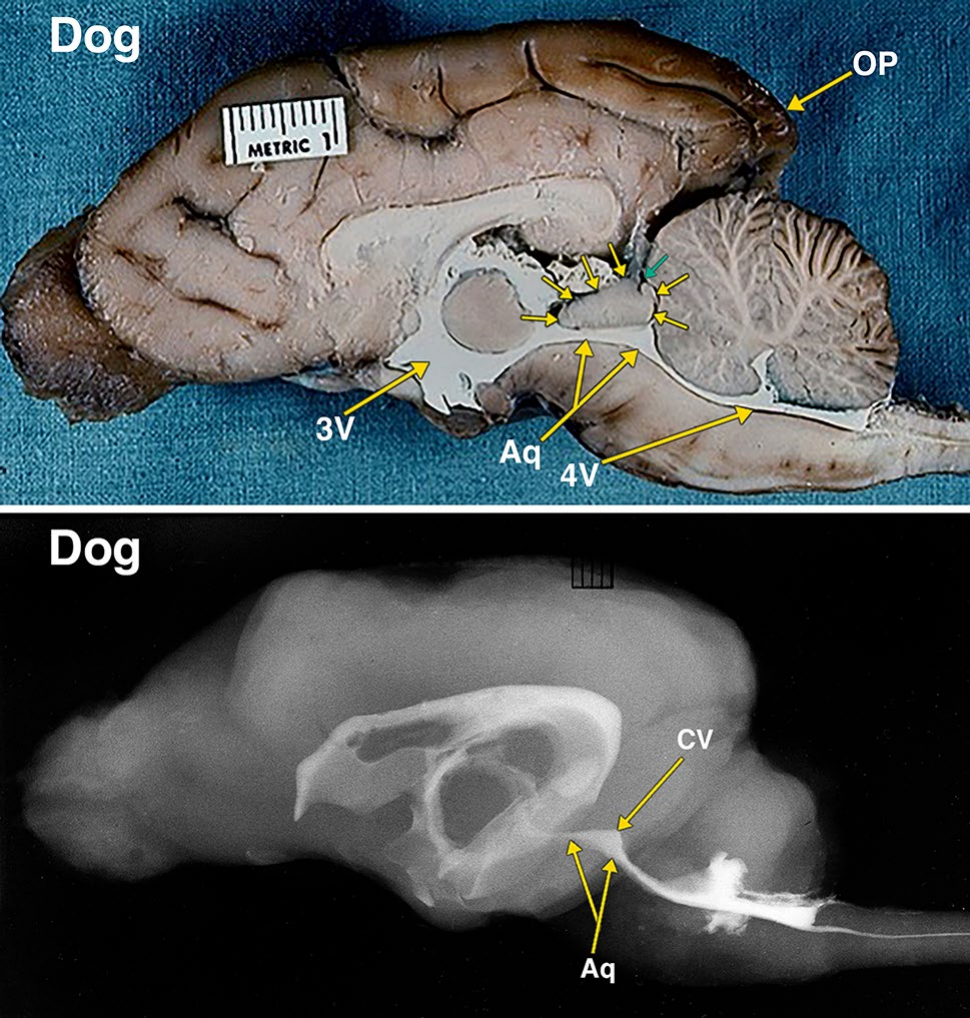
Dissected brain of a dog with radiopaque contrast filled cerebral ventricles. The midsagittal dissection (Fig. 6) demonstrates that the midbrain colliculi, outlined by multiple small arrows, is relatively similar in size when compared with cat in Fig. 5. However, the neocortical occipital lobe and pole (OP) when compared to the cat in Fig. 5 appear to extend further posteriorly of the cerebellum. A small cleft, indicated by a small green arrow, likely indicates the separate superior and inferior colliculi of the quadrigeminal plate. The collicular ventricle (CV) with its superior extensions features and its continuity with the midbrain aqueduct (Aq) are demonstrated in both the dissected specimen and the lateral radiograph. Additional abbreviations: 3 V, 3^d^ ventricle; 4 V, 4^th^ ventricle. The distance between 2 black lines on the ruler in the specimen photograph is 1 mm

**Fig. 7:**
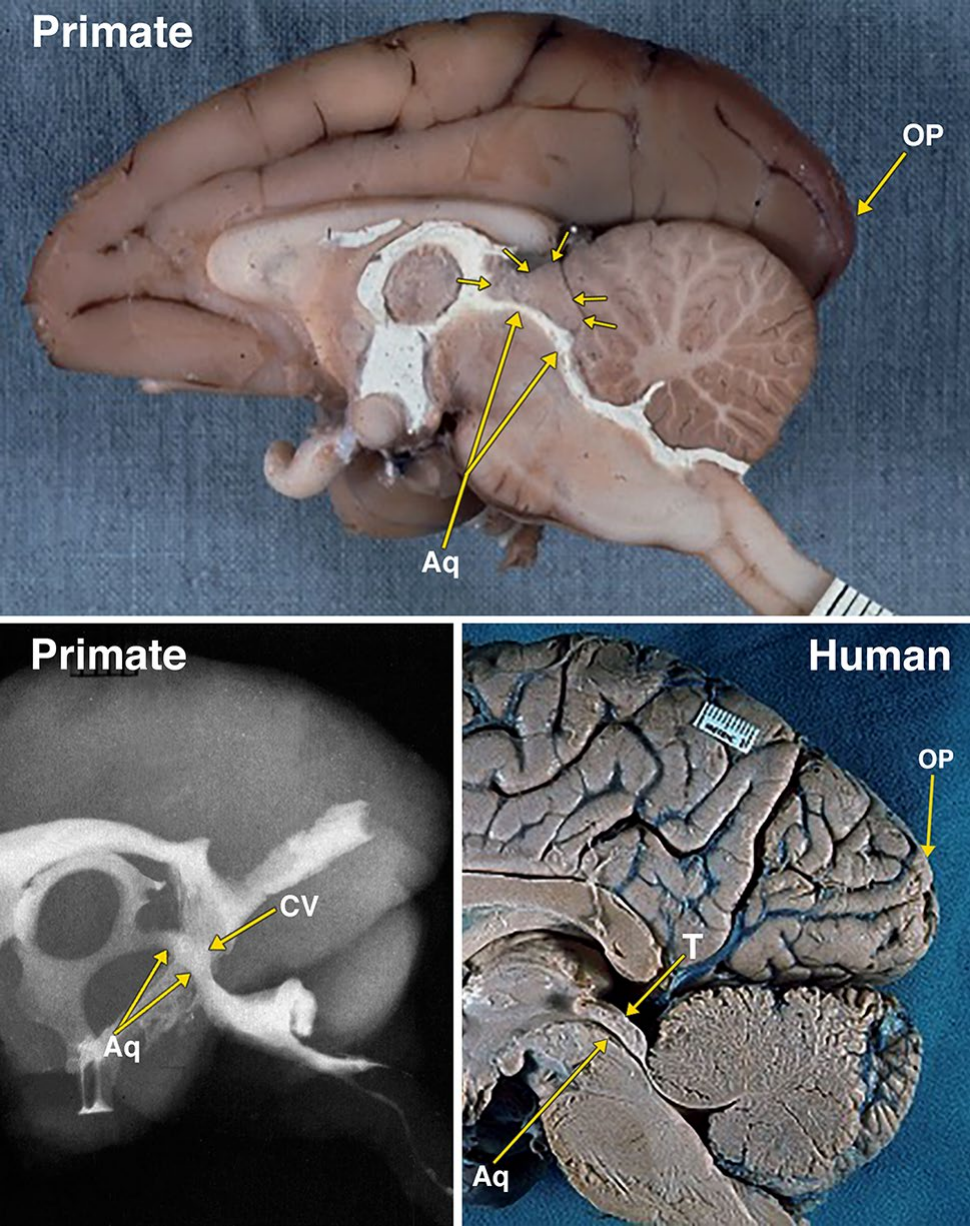
Dissected brain of a primate (macaque) with radiopaque contrast filled cerebral ventricles and a midsagittal adult human brain specimen. The midsagittal dissection (Fig. 7) of the primate demonstrates the midbrain colliculi, outlined by multiple small arrows, that are relatively smaller in size when compared with dog in Fig. 6. The small collicular ventricle (CV) can be identified on the midsagittal dissection and the radiograph. On the radiograph, a segment of the aqueduct (Aq) is not filled with the radiopaque contrast. The larger occipital lobe and pole (OP) extending further posteriorly above the cerebellum when compared to the dog in Fig. 6. Comparison of the primate with an adult human brain specimen, without intraventricular radiopaque contrast material, demonstrates the aqueduct (Aq), the relatively smaller tectum (T), and the marked expansion of the occipital lobe and pole (OP) in the human. The distance between 2 black lines on the rulers in the specimen photographs is 1 mm

### Photograph of dissections demonstrating contrast filled cerebral arteries in the rabbit and cat

**Fig. 8:**
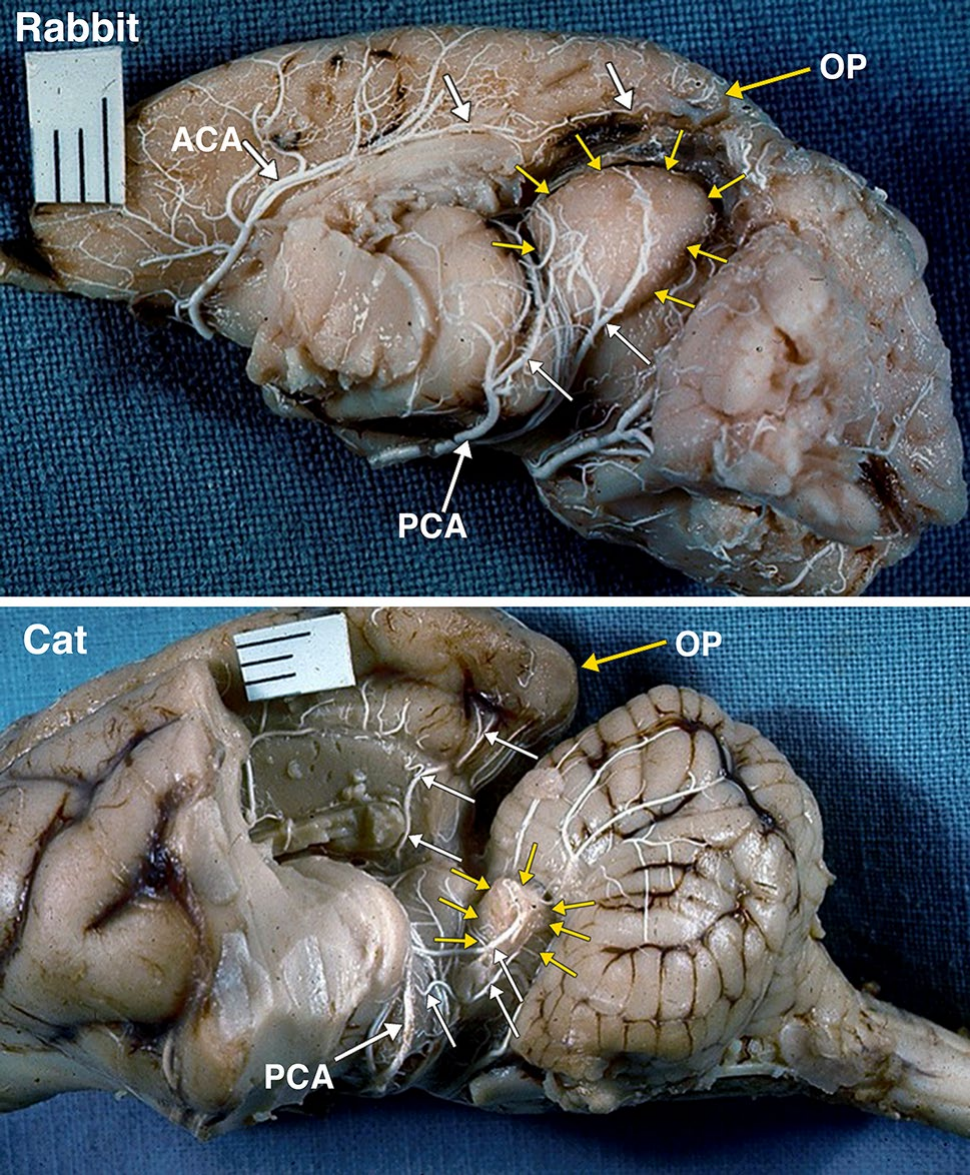
A postero-lateral view of an arterially injected and dissected left hemisphere of the brain of an adult rabbit. Identified are the large midbrain colliculi, outlined by yellow arrows. The colliculi are supplied by branches (white arrows) of the posterior cerebral artery (PCA). Of note is the small size of the occipital lobe and pole (OP) supplied by branches (white arrows) of the anterior cerebral artery (ACA). A postero-lateral view of an arterially injected and dissected left hemisphere of the brain of an adult cat. When compared to the rabbit (Fig. 8), the midbrain colliculi, outlined by yellow arrows are much reduced in size. The significant reduction in the relative size of colliculi is accompanied by significant increase in size of the occipital lobe and occipital pole (OP). The branches (white arrows) of the posterior cerebral artery (PCA) supply the colliculi, and also a large region of the occipital lobe. It was not possible to separately identify the superior and inferior colliculi without disrupting the arterial supply embedded in the arachnoid layer

In a lateral view of an arterially injected and dissected brain of a cat (Fig. 8), when compared to the rabbit (Fig. 8), the colliculi are relatively much smaller. The reduction in the size of the colliculi is accompanied by significant increase in size of the occipital lobe. Branches of the posterior cerebral artery supply the colliculi, and also a large region of the occipital lobe.

## Discussion

*Cephalization* is a key major evolutionary modifier of the brain [3, 25] and its ventricular and vascular systems. Telencephalization is a rarely used term that also describes the process of *cephalization* [10, 29].

Structurally advanced invertebrates and all vertebrates move head first, and as a result, the anterior region of the body is the first to meet environmental stimuli. Consequently, the placement of the major sense organs and their control at the most anterior (cranial) portion is advantageous. The process of cephalization consists of development of specialized sense organs, enlargement of the brain, and eventually the anterior shift of the control center to the forebrain.

A constant cephalic shift of the control centers of the nervous system took place throughout the entire evolutionary process of the vertebrate brain, and the most anterior segment of the brain became the most highly elaborated. This cephalic shift of control to the forebrain provided increased flexibility of function by the development of additional and more complex associative capacities. The process of cephalization is demonstrated in this investigation with the relative reduction in size of the midbrain tectum and increase in size of the occipital neocortex. This maybe the result of the majority of the optic fibers, instead of reaching the optic tectum, pass via the thalamus to the enlarging neocortical occipital lobe and adjacent temporal and parietal regions.

A disadvantage of cephalization is the loss of phylogenetically older control centers [25]. For instance, in the opossum, vision is maintained after damage to the cerebral cortex, whereas a similar lesion in humans causes blindness.

The vesicular brain undergoes pronounced evolutionary modifications in the development of the brain in fish, amphibian, reptilian, and mammalian, including primate and human stages. At each stage, the various components of the brain enlarge or diminish, depending on the specific needs of each species [12]. The ventricular system, which undergoes similar modifications, extends into new cerebral structures and changes as the various components of the brain enlarge or diminish.

### Evolution and embryology of the optic ventricles, mesencephalic ventricle, and aqueduct

The human cerebral aqueduct is a residual cavity which results from phylogenetic and embryologic modification of the walls surrounding the ventricular system of the midbrain. In the comparative anatomic literature, the term aqueduct is used to describe the midbrain ventricular cavity in all fishes, amphibians, and amniotes [22, 23]. The evolutionary changes of the tectum (roof) of the midbrain are complex and involve the optic lobes and the colliculi of the quadrigeminal plate.

The midbrain tectum is the primary visual center in nonmammalian vertebrates. The presence of the visual center in the tectum would seem to have been linked to the evolution of this region into a major association center [23]. The tectum in fishes and amphibians is the dominant brain center; it associates visual, olfactory, lateral line, and other somatic sensory stimuli with the motor columns of the brainstem and spinal cord.

In the *lamprey*, the nerve structures of the eye are developed and the optic tract ends in a discernable optic lobe [2]. Although it is still partly ependymal, it already has enlarged into a pair of recognizable optic lobes. The two optic lobes (corpora bigemina) are the homologues of the superior colliculi.

In the shark and the frog, the optic lobes are dominant structures of the brain. In the iguana, a reptile, the neocortical cerebrum is beginning to form and the optic lobes are relatively reduced in size. In some reptiles, a pair of auditory lobes, the homologues of the inferior colliculi, are present caudal to the optic lobes. The auditory lobes, which receive fibers from the cochlea, are not present in fishes that lack a cochlea. In amphibians, the cochlea is small and the auditory center is not large enough to form a bulge on the surface of the mesencephalon [13].

At some critical evolutionary stage. a supra-segmental area, more elaborate than the optic lobes, became necessary for the integration of refined visual perceptions. [3, 25, 27] The evolution of the cerebrum provided a more elaborate supra-segmental region capable of integrating refined visual perceptions such as color, form, size, and detection of motion and distance. In mammals, most of the visual, auditory, and other somatic sensations, instead of being integrated in the midbrain, are relayed via the thalamus to the cerebral hemispheres. The thalamus in nonmammalian vertebrates is a small anterior extension of the sensory association area of the midbrain. It reaches its maximum development in mammals as a result of its function as a relay center to the association centers in the cerebral hemispheres. 

The differentiation of the optic lobe tectum to a tectum consisting of the superior and inferior coliculli (corpora quadrigemina) occurs only in mammals [29]. The cephalic shift in mammals demonstrated in this investigation is the changes in the sizes of the cerebrum and the colliculi. The increase in the size of the neocorical occipital lobe from the rabbit to the human is associated with a relative reduction in the size of the corpora quadrigemina (Figs. 4–8).

Previously, the superior colliculi were considered to be exclusively an optic structure and thus the homologue of the optic lobes. The inferior colliculi were considered to be auditory centers which developed as a result of the evolution of the hearing function of the ear. This interpretation of collicular function has been modified as other connections than purely visual and auditory have been demonstrated in the colliculi.

Several publications have summarized the complex extensive new research dealing with what is referred to as the optic tectum, and other new concepts and research regarding evolution, phylogeny, and homology [4, 10, 11, 21, 25, 28, 31].

As demonstrated in this investigation, the aqueduct and the optic and collicular ventricles form a continuous cavity. Thus, it is likely that the absence of the remnants of the optic and collicular ventricles in the human tectum are the result of the reduced relative size of the human tectum, and/or the result of additional or rearranged fiber tracts in the tectum.

Another possibility to consider is that the aqueductal ampulla, the widest segment of the human aqueduct, is a homologous remnant of the optic and collicular ventricles. The studies that provided measurement of the various segments of the human aqueduct indicate that the ampulla has a larger volume than the other sections of the aqueduct [8, 20, 30]. These studies do not indicate that the ampulla has an increased height. A neuroendoscopic view of the ampulla shows it as a horizontal ellipse [19]. There is the possibility that the enlargement of the periaqueductal tissue in the human changed the increased vertical height to a horizontal one.

## Summary

This investigation emphasized the role of vertebrate anatomic dissections and radiography in helping introduce comparative anatomic changes in expanding the list of potential explanations regarding non-tumoral aqueductal stenosis.

The midbrain tectum, consisting of the optic lobes, optic ventricles, colliculi, and collicular ventricles, undergoes evolutionary cephalization modifications resulting in relative tectum size reduction, aqueductal changes, associated with enlargement of the occipital lobe, and posterior cerebral artery distribution modifications. Potentially, these evolutionary modifications may explain aqueductal anomalies such as forking, septa, membranes, and stenosis in humans.

The evolutionary modifications resulting in diminution in the size of the midbrain tectum, and tectal ventricles, may result in aqueductal reduced diameter. The reduction in tectal size and possible reduction in aqueductal diameter may reduce the resistance of the aqueduct to the compressive effects of the periaqueductal structures. All these may be potential factors contributing towards aqueductal stenosis in humans.
